# A Systematic Review of the Metabolism of High-Grade Gliomas: Current Targeted Therapies and Future Perspectives

**DOI:** 10.3390/ijms25020724

**Published:** 2024-01-05

**Authors:** Lucio De Maria, Pier Paolo Panciani, Marco Zeppieri, Tamara Ius, Simona Serioli, Amedeo Piazza, Emanuele Di Giovanni, Marco Maria Fontanella, Edoardo Agosti

**Affiliations:** 1 Division of Neurosurgery, Department of Medical and Surgical Specialties, Radiological Sciences and Public Health, University of Brescia, Piazza Spedali Civili 1, 25123 Brescia, Italy or lucio.demaria@hcuge.ch (L.D.M.); edoardo_agosti@libero.it (E.A.); 2 Division of Neurosurgery, Department of Clinical Neurosciences, Geneva University Hospitals (HUG), Rue Gabrielle-Perret-Gentil 4, 1205 Geneva, Switzerland; 3 Department of Ophthalmology, University Hospital of Udine, p.le S. Maria della Misericordia 15, 33100 Udine, Italy; 4 Neurosurgery Unit, Head-Neck and NeuroScience Department University Hospital of Udine, p.le S. Maria della Misericordia 15, 33100 Udine, Italy; 5 Department of Neurosurgery, “Sapienza” University, 00185 Rome, Italy

**Keywords:** high-grade gliomas, metabolism, target therapies, survival

## Abstract

High-grade glial tumors (HGGs) exhibit aggressive growth patterns and high recurrence rates. The prevailing treatment approach comprises radiation therapy (RT), chemotherapy (CMT), and surgical resection. Despite the progress made in traditional treatments, the outlook for patients with HGGs remains bleak. Tumor metabolism is emerging as a potential target for glioma therapies, a promising approach that harnesses the metabolism to target tumor cells. However, the efficacy of therapies targeting the metabolism of HGGs remains unclear, compelling a comprehensive review. This study aimed to assess the outcome of present trials on HGG therapies targeting metabolism. A comprehensive search of PubMed, Ovid MEDLINE, and Ovid EMBASE was conducted until November 2023. The search method used pertinent Medical Subject Heading (MeSH) terminologies and keywords referring to “high-grade gliomas”, “metabolism”, “target therapies”, “monoclonal antibodies”, “overall survival”, and “progression-free survival”. The review analyzed studies that focused on therapies targeting the metabolism of HGGs in human subjects. These studies included both randomized controlled trials (RCTs) and non-randomized controlled trials (NRCTs). Out of 284 articles identified, 23 trials met the inclusion criteria and were thoroughly analyzed. Phase II trials were the most numerous (62%). Targeted metabolic therapies were predominantly used for recurrent HGGs (67%). The most common targeted pathways were the vascular endothelial growth factor (VEGF, 43%), the human epidermal growth factor receptor (HER, 22%), the platelet-derived growth factor (PDGF, 17%), and the mammalian target of rapamycin (mTOR, 17%). In 39% of studies, the subject treatment was combined with CMT (22%), RT (4%), or both (13%). The median OS widely ranged from 4 to 26.3 months, while the median PFS ranged from 1.5 to 13 months. This systematic literature review offers a thorough exploration of the present state of metabolic therapies for HGGs. The multitude of targeted pathways underscores the intricate nature of addressing the metabolic aspects of these tumors. Despite existing challenges, these findings provide valuable insights, guiding future research endeavors. The results serve as a foundation for refining treatment strategies and enhancing patient outcomes within the complex landscape of HGGs.

## 1. Introduction

Gliomas are brain tumors originating from glial or neural precursor cells, representing 80.9% of central nervous system (CNS) primary malignant tumors in adults [1]. In recent years, research has increasingly refined conventional combined treatments such as surgery, chemotherapy (CMT), and radiotherapy (RT), thanks to the introduction of the Stupp protocol in 2005 [2]. However, nowadays, high-grade glial tumors (HGGs) are still characterized by poor results in terms of prognosis, with a mean survival of 18 months, due to their complex tumoral heterogeneity [3,4]. The role of inter- and intra-tumoral heterogeneity, based on microenvironmental features and genetic and epigenetic expression, combined with elevated mitotic activity and microvascular and brain invasion, represents a crucial factor for this subgroup of tumors, influencing the response to treatments [5]. Furthermore, conventional MRI does not allow the identification of those infiltrative tumor cells in the peritumoral area, limiting the effectiveness of surgical resection and radiation and determining a high risk of recurrence and rapid progression [6].

The introduction of genomic analysis has offered a fundamental contribution to histologic interpretation, identifying different tumoral subgroups. In 2008, the Cancer Genome Atlas (TCGA) Research Network allowed for the first time a broad analysis of the glioblastoma (GBM) genome, highlighting the main aberrations and cancer driver mutations and identifying four major molecular subgroups (proneural, neural, classical, and mesenchymal transcriptomics) [7]. Moreover, the phosphatase and tensin homolog (PTEN) gene mutation, whose deficiency is associated with poor prognosis, has been documented in 35% of GBMs. Conversely, the activation of receptor tyrosine kinase (RTK)/phosphoinositide 3-kinase (PI3K) has been found in 86% of the cases. The identification of the isocitrate dehydrogenase (IDH) 1 mutation has been shown to make tumor cells more vulnerable to radio-chemotherapy, thanks to the reduced activity of the nicotinamide adenine dinucleotide phosphate (NADPH) enzyme, with a relevant prognostic effect and improved quality of life in IDH-mutated gliomas [8].

Considering their role in diagnosis and prognosis, specific genomic variations, including IDH, histone 3-3A (H3-3A), or telomerase reverse transcriptase (TERTp) mutation, and chromosome remodeling, such as 1p/19q codeletion, combined chromosome 7 gain and chromosome 10 loss, O-6-methylguanineDNA methyltransferase (MGMT) gene methylation, and epidermal growth factor receptor (EGFR) amplification [9,10,11], were largely integrated into the 2021 fifth edition of the WHO classification [12]. However, molecular target therapies have provided limited results due to inter- and intra-tumoral variability, disease progression/relapse, and therapeutic failure [13,14].

In recent years, there has also been growing interest in immunotherapy, considering the promising results obtained with other types of tumors. Immune checkpoints, vaccines, oncolytic immunovirotherapy, and CAR T-cell therapy, combined with conventional treatments, could improve clinical outcomes. However, the tumor immunosuppressive action and the brain-blood barrier (BBB) are elements that cannot be ignored, and further clinical studies are necessary to fully understand the potential of this treatment in HGGs [15].

Recently, the glioma metabolic mechanisms and their relationship with the tumoral microenvironment have acquired importance, especially for the possibility of targeting essential key metabolic enzymes, redox homeostasis, and cancer-stem-like cells (CSC) [16,17,18]. This population of cells, characterized by self-renewal and metabolic dynamism, is a crucial factor in disease progression and recurrence in HGGs [17,19]. Several studies have highlighted how the upregulation of factors such as Oct4, Nanog, SOX-2, and c-Myc in hypoxia and chemo-radiation treatments, and the hyperactivation of the tyrosine kinase c-Met or activation of epithelial-to-mesenchymal transition (EMT) can trigger bidirectional plasticity between CSCs and differentiated tumor cells [20,21,22,23,24]. Furthermore, CSCs can self-regulate essential key metabolic enzymes based on the availability of oxygen and metabolites (glucose, pyruvate, and lactate) to ensure their cell survival as the conditions of the tumor microenvironment (TME) change, acting on the oxidative and non-oxidative metabolism of glucose, mitochondrial oxidative metabolism, glutamine, and lipid metabolism of CSCs [21].

Understanding the altered metabolic pathways could offer new therapeutic options in the complex management of HGG, proposing personalized treatments to be integrated with traditional therapies to exploit the synergistic combination of multiple treatments. Several metabolic therapies are emerging that involve pathways such as vascular endothelial growth factor receptor (VEGFR), human epidermal growth factor receptor (HER), platelet-derived growth factor (PDGF), and mammalian target of rapamycin (mTOR). The present study offers a comprehensive overview of the clinical trials on current therapies targeting metabolism for HGGs, highlighting the efficacy of these novel treatments and the initial clinical results for future therapeutic perspectives.

## 2. Results

### 2.1. Literature Review

**Figure 1:**
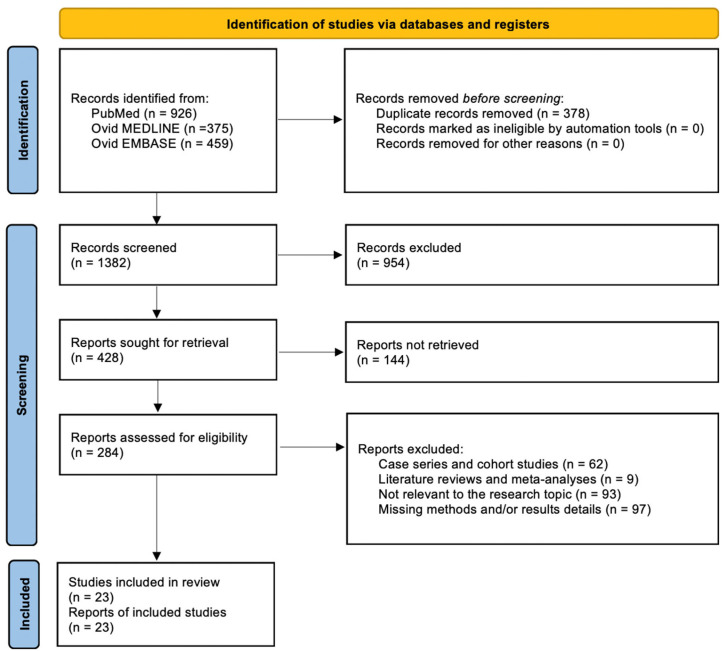
PRISMA flow chart.

A total of 1382 papers were identified after duplicate removal. After title and abstract analysis, 428 articles were identified for full-text analysis. Out of 284 articles, eligibility was evaluated for all of them, and only 23 articles met the criteria. The remaining 261 articles were excluded for the following reasons: 62 articles were case series and cohort studies; 9 articles were systematic literature reviews or meta-analyses; 93 articles were not relevant to the research topic; and 97 articles lacked sufficient details on methods and/or results. All studies included in the analysis had at least one or more outcome measures available for one or more of the patient groups analyzed. Figure 1 shows the flow chart according to the PRISMA statement.

**Figure A1:**
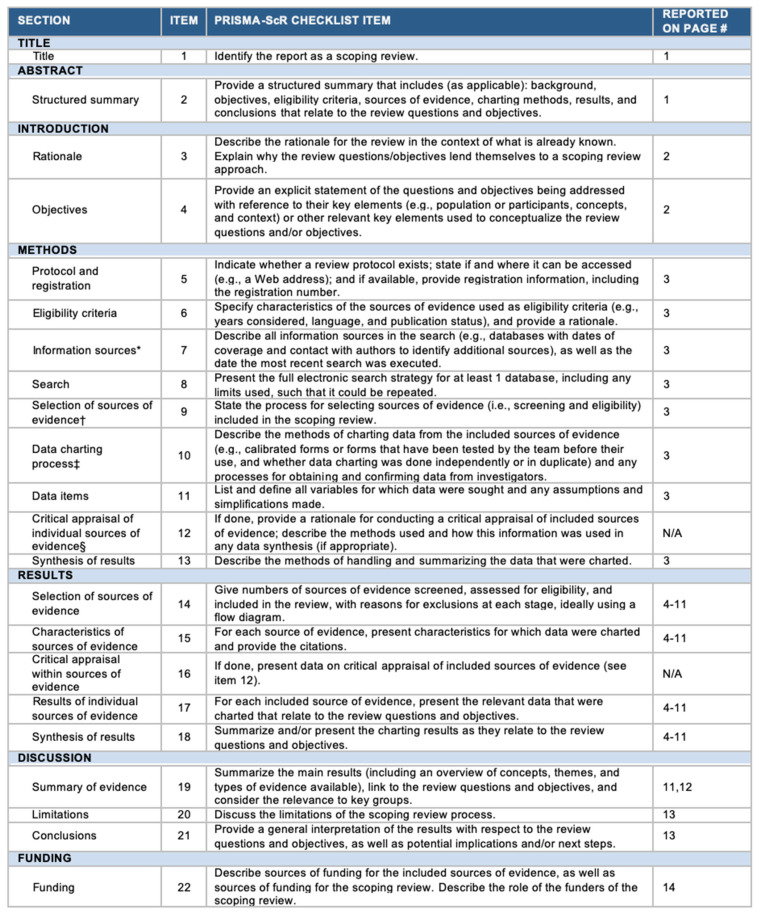
PRISMA-ScR checklist. * Where sources of evidence (see second footnote) are compiled from, such as bibliographic databases, social media platforms, and Web sites. † A more inclusive/heterogeneous term used to account for the different types of evidence or data sources (e.g., quantitative and/or qualitative research, expert opinion, and policy documents) that may be eligible in a scoping review as opposed to only studies. This is not to be confused with information sources (see first footnote). ‡ The frameworks by Arksey and O’Malley and Levac and colleagues and the JBI guidance refer to the process of data extraction in a scoping review as data charting. § The process of systematically examining research evidence to assess its validity, results, and relevance before using it to inform a decision. This term is used for items 12 and 19 instead of "risk of bias" (which is more applicable to systematic reviews of interventions) to include and acknowledge the various sources of evidence that may be used in a scoping review (e.g., quantitative and/or qualitative research, expert opinion, and policy document). N/A not available.

The PRISMA Extension for Scoping Reviews (PRISMA-ScR) checklist is available in Appendix A (Figure A1).

### 2.2. Extracted Data

Table 1 provides an overview of the studies that have been included.

The systematic review that we conducted only consisted of clinical trials that were carried out between 2005 and 2022. The total number of patients enrolled in the trials was 4980. Among the scientific journals that published these trials, *Neuro-Oncology*, the *Journal of Neuro-Oncology*, and the *Journal of Clinical Oncology* had the highest number of publications (9/23, 39%). *Clinical Cancer Research*, *Cancer*, and the *British Journal of Cancer* followed the lead with 6 out of 23 (27%). The majority of these studies were published between 2015 and 2022 (13/23, 57%).

Out of the studies conducted, the majority (62%) were Phase II trials, followed by Phase I trials (29%), while only a small proportion (10%) comprised Phase III trials. In the majority of cases (65%), targeted metabolic therapies were utilized for treating recurrent HGGs. On the other hand, in 35% of the cases, they were employed as the primary treatment. The targeted cohort was only comprised of WHO grade IV gliomas in the majority of trials (74%), and a combination of both WHO grade IV and grade III gliomas in the remaining trials (6%).

**Figure 2:**
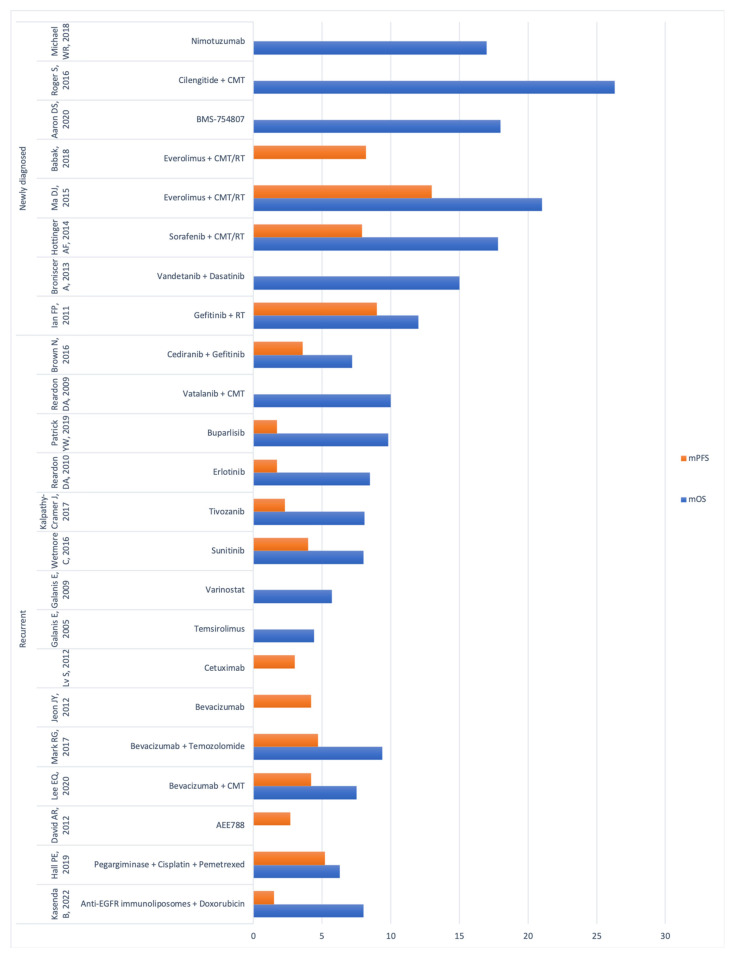
Bar plot displaying median OS (mOS) and median PFS (mPFS) per each trial included in the study [25,26,27,28,29,30,31,32,33,34,35,36,37,38,39,40,41,42,43,44,45,46,47].

Concerning the classes of metabolic therapeutic agents, the most common treatment targets were the VEGF pathway (10/23, 43%), the HER pathway (5/23, 22%), the PDGF pathway (4/23, 17%), and the mTOR pathway (4/23, 17%). Other less common targeted pathways were receptor tyrosine kinase (RTK), rearranged during transfection (RET), and others. The majority of the studies included (14/23, 61%) focused only on metabolic treatment as the primary therapy, without supplementing it with other adjuvant therapies like radiation therapy (RT) or chemotherapy (CMT). In the remaining 9 studies (39%), the studied treatment was combined with CMT (5/23, 22%), RT (1/23, 4%), or both (3/23, 13%). The median OS widely ranged from 4 to 26.3 months, while the median PFS ranged from 1.5 to 13 months. A graphical representation of the main outcomes of the study is shown in Figure 2.

A summary of the current trials on HGG metabolic therapies grouped by target pathway is presented in Table 2, Table 3, Table 4 and Table 5.

## 3. Discussion

The challenging landscape of HGGs demands innovative therapeutic approaches. In recent years, substantial progress has been made in understanding the molecular underpinnings of these tumors, leading to the identification of specific genomic alterations and chromosomal remodeling. The integration of these molecular aspects into the WHO classification underscores their significance in diagnosis and prognosis. However, molecular target therapies, despite their promise, face challenges such as intra-tumoral variability, disease progression, relapse, and therapeutic failure.

The systematic literature review presented here aimed to provide a comprehensive overview of clinical trials focusing on metabolic therapies for HGGs, emphasizing the efficacy of these treatments, and exploring future perspectives. The inclusion criteria ensured a rigorous selection of studies, encompassing clinical trials in English that specifically targeted cellular metabolism in WHO grade III and grade IV gliomas, with a focus on OS and PFS as primary outcomes.

### 3.1. Overview of Included Studies

A meticulous literature search identified 23 clinical trials meeting the inclusion criteria, enrolling a total of 4980 patients. These trials spanned from 2005 to 2022, with the majority published between 2015 and 2022, indicating a recent surge in research interest. Noteworthy journals such as *Neuro-Oncology*, the *Journal of Neuro-Oncology*, and the *Journal of Clinical Oncology* published the highest number of these trials.

The predominant trial design was Phase II (62%), reflecting the exploratory nature of metabolic therapies in HGGs. Targeted metabolic therapies were frequently employed for recurrent HGGs (65%), highlighting the urgent need for effective treatments in cases of disease recurrence. Most trials focused on WHO grade IV gliomas (74%), with a smaller subset including both grade IV and grade III gliomas (6%).

### 3.2. Targeted Pathways and Therapeutic Classes

The review identified four major metabolic pathways as targets in the included studies: the VEGF pathway (43%), the HER pathway (22%), the PDGF pathway (17%), and the mTOR pathway (17%). Less common pathways included RTK, RET, and others.

Most studies (61%) investigated targeted metabolic treatments as standalone therapies, emphasizing the potential of these interventions to exert meaningful effects independently. In the remaining studies (39%), the metabolic treatments were combined with CMT, RT, or both. This combination approach may offer synergistic benefits, addressing the complex heterogeneity of HGGs.

### 3.3. Individual Targeted Pathways

VEGF Pathway. Clinical trials targeting the VEGF pathway, such as those employing bevacizumab, cediranib, and tivozanib, demonstrated varied outcomes in terms of OS and PFS. Notably, bevacizumab, a monoclonal antibody against VEGF, showed promise in several studies, aligning with findings from previous research [27,36,42]. Cediranib, a VEGFR inhibitor, exhibited mixed results, emphasizing the complexity of targeting this pathway [38]. The heterogeneity in patient populations, treatment regimens, and trial designs may contribute to the observed variations in outcomes.

HER Pathway. Studies targeting the HER pathway, including nimotuzumab, cetuximab, gefitinib, and AEE788, provided insights into the potential of these agents. Nimotuzumab, an EGFR antibody, exhibited positive OS and PFS outcomes, suggesting its relevance in HGG treatment [30]. Similarly, other agents targeting EGFR, such as cetuximab and gefitinib, demonstrated varying efficacy, underscoring the importance of patient selection and individualized treatment strategies [39,45]. AEE788, targeting both EGFR and ERBB2, showed promise in terms of PFS, indicating the potential benefit of dual pathway inhibition [44].

PDGF Pathway. Clinical trials focusing on the PDGF pathway, utilizing sorafenib, sunitinib, and vatalanib, presented diverse outcomes. Sorafenib, a multikinase inhibitor, demonstrated notable OS and PFS benefits, suggesting its potential role in HGG management [48]. Sunitinib, a VEGFR and PDGFR inhibitor, exhibited mixed results, indicating the need for further investigation and potentially refined patient selection [29]. Vatalanib, a VEGFR inhibitor, showed positive OS outcomes, highlighting the potential impact of PDGF pathway targeting [40].

mTOR Pathway. Trials targeting the mTOR pathway with agents like erlotinib, everolimus, temsirolimus, and BMS-754807 provided valuable insights. Erlotinib, an EGFR inhibitor with mTOR inhibitory effects, demonstrated promising OS and PFS results [33]. Everolimus, an mTOR inhibitor, exhibited mixed outcomes, emphasizing the need for careful patient selection and potential combination therapies [37,43]. Temsirolimus, another mTOR inhibitor, presented positive OS outcomes, indicating the potential efficacy of mTOR pathway inhibition [41]. BMS-754807, targeting IGF1R, demonstrated potential benefits in terms of OS, underlining the significance of exploring alternative pathways [46].

### 3.4. The Role of Tumor Microenvironment and Immunotherapy

Despite recent advances in combined treatments, HGGs have been challenging to treat, and the prognosis has remained poor. The complex microenvironment of glial tumors has a significant impact on the effectiveness of therapies [5]. However, recent studies have shown that immunotherapy could be a promising strategy to control TME and improve clinical outcomes significantly [49]. Combining conventional treatments with immune checkpoints, vaccines, oncolytic immunovirotherapy, and CAR T-cell therapy has shown great potential for treating HGGs. CAR T-cell therapy, in particular, has emerged as a promising modality in glioblastoma immunotherapy, with recent advancements in second-generation CAR T-cells that offer heightened specificity and fewer off-target effects. Studies targeting antigens such as IL13Rα2, EGFRvIII, and HER2 have shown remarkable tumor regression and improved survival rates in patients [50,51]. Although some challenges persist, the concept of developing trivalent CAR T-cells that target multiple antigens at once offers hope for even more effective treatment outcomes [52,53,54,55,56,57,58,59,60,61,62,63,64,65,66].

### 3.5. Challenges and Future Directions

Although clinical trials have provided important insights, there are still several challenges that need to be addressed. One such challenge is inter- and intra-tumoral variability, disease progression, and therapeutic resistance, which continue to be significant hurdles. Metabolic adaptation is a factor to be taken into account, which can play a role in resistance to therapies targeting metabolism at the molecular level. This phenomenon has been observed for mTOR inhibitors and anti-angiogenic therapies targeting the VEGFR pathway [41,67]. The differences in patient populations, tumor heterogeneity, and trial designs may contribute to the observed variability in outcomes across trials. Furthermore, the lack of standardized approaches and biomarkers for patient stratification makes it difficult to identify responders. It is also important to note that the long-term impact of targeted metabolic therapies on HGG patients is not yet fully understood. The current studies do not have sufficient follow-up results that span a significant period. Therefore, the extended effectiveness and benefits of innovative metabolic therapies are still uncertain, and any possible negative effects that may occur later on are not clear.

Future research directions should address these challenges and explore novel avenues for improving the efficacy of metabolic therapies in HGGs. Personalized treatment approaches, guided by molecular profiling and predictive biomarkers, could enhance treatment outcomes. Combining metabolic therapies with conventional treatments, such as CMT and RT, warrants further investigation to harness potential synergies. Additionally, future studies should explore the potential benefits of combining inhibitors of multiple metabolic targets at the same time to overcome therapeutic resistance, such as metabolic adaptation. To illustrate, blocking glutamines could make GBM cell lines and xenografts more responsive to mTOR inhibitors, as per some studies [68,69]. Similarly, pyruvate dehydrogenase activators like dichloroacetate have been shown to potentially enhance the effectiveness of VEGFR inhibitors [70,71]. These combinations may be worthy of clinical investigation in future clinical trials on HGG metabolic therapies. In addition, upcoming clinical studies need to examine the possible adverse impacts in the long run, and the actual advantages of these treatments should be established by conducting extended follow-up clinical trials.

## 4. Methods

### 4.1. Review of the Literature

The authors followed the Preferred Reporting Items for Systematic Reviews and Meta-Analysis (PRISMA) guidelines [72]. A comprehensive literature search of PubMed, Ovid MED-LINE, and Ovid EMBASE was conducted using a combination of keyword searches. The search was performed twice, once on 29 September 2023, and then updated on 19 November 2023. The search terms that were utilized in both AND and OR combinations include “high-grade glioma”, “metabolism”, “targeted therapy”, “monoclonal antibodies”, “overall survival”, and “progression-free survival”. The following combination of Medical Subject Heading (MeSH) terms and Boolean operators was used: (high-grade glioma OR HGG OR glioblastoma OR GBM OR GBL) AND metabolism AND target therapy AND outcomes AND (prognosis OR progression-free survival OR overall survival). The search filter was configured to display publications that fall within the specified time frame of 2000 to 2023. The authors used a set of inclusion and exclusion criteria to select studies that met their requirements. The criteria for inclusion were as follows: (1) English language; (2) clinical trials; (3) studies on World Health Organization (WHO) grade III and grade IV gliomas; (4) studies on targeted therapies focused on cellular metabolism; and (5) studies including overall survival (OS) or progression-free survival (PFS) among the outcomes analyzed. The following studies were excluded: (1) editorials, case reports and case series, cohort studies, reviews, and meta-analyses; (2) studies not well-defining methods and results; and (3) studies not reporting data on PFS or OS. By analyzing the references of selected papers, more pertinent articles were identified.

The list of studies was imported into Endnote X9, and any duplicates were eliminated. The results were checked by two independent researchers (L.D.M. and E.A.) according to the inclusion and exclusion criteria. All disagreements were resolved by a third reviewer (M.Z.). Finally, the eligible articles were subjected to full-text screening.

### 4.2. Data Collection

We collected the following details for each study: authors, publication year and journal, clinical trial’s name and phase, patient count, diagnosis, follow-up duration, outcomes, and treatment type.

### 4.3. Objectives

The main focus of our study was to evaluate the effectiveness of targeted metabolic therapies in terms of OS and PFS for patients diagnosed with HGGs.

### 4.4. Evaluation of the Potential for Bias

In order to evaluate the quality of the studies included in the research, the Newcastle-Ottawa Scale (NOS) was utilized. The assessment of the quality was based on the selection criteria, comparability of the study, and outcome assessment. The score ranged from 0 to 9, with higher scores indicating better quality of studies. To be considered high-quality studies, they had to receive 7 or more points. The quality assessment was performed by two authors (M.M.F. and P.P.P.) independently. If any discrepancies arose, a third author re-examined the papers (Table 6).

### 4.5. Statistics

The statistical analysis used in our study involved the use of various descriptive statistics, such as sums, proportions, percentages, and ranges. These measures are commonly used to summarize and describe data, and they can provide insights into the data’s characteristics. The analysis was carried out using the R statistical package v3.4.1 1 (http://www.r-project.org, accessed on 10 September 2023), which is a software tool used for statistical computing and graphics. R offers a wide range of functions that enable data visualization and analysis, allowing one to model data as required and create graphics. Additionally, several modules enhance the software’s graphical functions, making it a versatile tool for data analysis and visualization. Overall, the use of R for statistical analysis is becoming increasingly popular among researchers and analysts due to its versatility and ease of use. The descriptive statistics described in the text are just one example of the many statistical tools available in R, and users can tailor their analyses to meet their specific needs.

## 5. Conclusions

This literature review presents a thorough analysis of the current landscape of metabolic therapies for HGGs. The complexity of addressing the metabolic aspects of these tumors is highlighted by the wide range of targeted pathways and therapeutic categories. However, four metabolic pathways, including the VEGF, HER, PDGF, and mTOR pathways, have been the focus of extensive research. Although clinical trials have provided critical insights, several challenges remain, including intra- and inter-tumoral variability, tumor heterogeneity, disease progression, and therapeutic resistance. Furthermore, the long-term impact of targeted metabolic therapies on HGG patients is not yet fully understood. Nevertheless, the results of these trials offer valuable insights and pave the way for future research aimed at refining treatment strategies and improving patient outcomes. There is potential to improve treatment outcomes through personalized approaches that use molecular profiling and predictive biomarkers. Investigating the synergies between metabolic therapies and traditional treatments like CMT and RT is worth continuing to explore. Additionally, it may be beneficial to examine the effects of using inhibitors of multiple metabolic targets simultaneously to overcome therapeutic resistance. Upcoming clinical studies should also assess any potential long-term adverse impacts, and extended follow-up clinical trials are needed to establish the actual benefits of these treatments.

## Figures and Tables

**Table 1:** Overview of the studies. NA: not available.

Author	Journal	Year	Phase	Patients (No.)	WHO Grade	Recurrent or Newly Diagnosed	Median OS (Months)	Median PFS (Months)	Treatment	Combination with CMT or RT or Both	Target
Kasenda B et al. [25]	*ESMO Open*	2022	I	9	IV	NA	8	1.5	Anti-EGFR immunoliposomes loaded with Doxorubicin	No	Telomerase II
Hall PE et al. [26]	*Clin Cancer Res*	2019	I	10	III–IV	Recurrent	6.3	5.2	Pegargiminase, Cisplatin, and Pemetrexed	CMT	Arginine
Lee EQ et al. [27]	*Cancer*	2020	II	57	IV	NA	7.5	4.2	Bevacizumab	Alone and with CMT	VEGF
Hottinger AF et al. [28]	*Br J Cancer*	2014	I	17	III–IV	Newly diagnosed	17.8	7.9	Sorafenib	Both	VEGFR, Flt3, c-RAF, wild type and V599E mutant B-RAF, PDGFRβ, c-KIT, FGFR1, p38α and RET
Wetmore C et al. [29]	*Cancer Med*	2016	II	30	III–IV	NA	8	4	Sunitinib	No	VEGFR, PDGFR, KIT
Michael W Ronellenfitsch et al. [30]	*Acta Neuropathol Commun*	2018	III	149	IV	Newly diagnosed	17	NA	Nimotuzumab	No	EGFR
Kalpathy-Cramer J et al. [31]	*J Neurooncol*	2017	II	10	IV	Recurrent	8.1	2.3	Tivozanib	No	VEGFR
Broniscer A et al. [32]	*Clin Cancer Res*	2013	I	25	IV	Newly diagnosed	15	NA	Vandetanib and Dasatinib	No	Dasatinib: c-Kit, Src, and PDGFRA and B; Vandetanib: VEGF receptor 2, EGFR and RET
Reardon DA et al. [33]	*J Neurooncol*	2010	II	32	IV	Recurrent	8.5	1.7	Erlotinib	No	mTOR
Roger Stupp et al. [34]	*The Lancet Oncol*	2016	III	3471	IV	Newly diagnosed	26.3	NA	Cilengitide	CMT	αvβ3 and αvβ5 integrin
Galanis E et al. [35]	*J Clin Oncol*	2009	II	66	IV	Recurrent	5.7	NA	Varinostat	No	Histone deacetylase
Mark R Gilbert et al. [36]	*J Neurooncol*	2017	II	60	IV	Recurrent	9.4 (temozolomide arm); 7.7 (ironotecan arm)	4.7 (temozolomide arm); 4.1 (irinotecan arm)	Bevacizumab	CMT	VEGF
Ma DJ et al. [37]	*Neuro Oncol*	2015	II	100	IV	Newly diagnosed	21	13	Everolimus	Both	mTOR
Brown N et al. [38]	*PLoS One*	2016	II	38	IV	Recurrent	5.5 (placebo); 7.2 (combined with gefitinib)	2.8 (placebo); 3.6 (combined with gefitinib)	Cediranib	No	VEGFR
Lv S et al. [39]	*Int J Oncol*	2012	II	35	IV	Recurrent	5.6 (EGFR amplification and EGFRvIII negative glioblastoma); 4 (EGFR amplification and EGFRvIII positive glioblastoma)	3 (EGFR amplification and EGFRvIII negative glioblastoma); 1.6 (EGFR amplification and EGFRvIII positive glioblastoma)	Cetuximab	No	EGFR
Reardon DA et al. [40]	*Cancer*	2009	I	37	III–IV	Recurrent	10	NA	Vatalanib	CMT	VEGFR, PDGFR
Galanis E et al. [41]	*J Clin Oncol*	2005	II	65	IV	Recurrent	4.4	NA	Temsirolimus	No	mTOR
Jeon JY et al. [42]	*Am J Neuroradiol*	2012	NA	18	IV	Recurrent	NA	4.2	Bevacizumab	No	VEGF
Babak et al. [43]	*Neuro Oncol*	2018	II	171	IV	Newly diagnosed	NA	8.2	Everolimus	Both	mTORC
David A. Reardon [44]	*Cancer Chemother Pharmacol*	2012	I	64	IV	Recurrent	NA	2.7 (arm 1); 1.6 (arm 2)	AEE788	No	ERBB2, VEGFR
Ian F. Pollack et al. [45]	*Neuro Oncol*	2011	II	43	III–IV	Newly diagnosed	12	9	Gefitinib	RT	EGFR
Aaron D Simpson et al. [46]	*Br J Cancer*	2020	NA	408	III–IV	Newly diagnosed	18	NA	BMS-754807	No	IGF1R
Patrick Y Wen et al. [47]	*J Clin Oncol*	2019	II	65	IV	Recurrent	17.9 (eligible for re-operation); 9.8 (not eligible for re-operation)	1.7	Buparlisib	No	PI3K

**Table 2:** Summary of the studies on HGG metabolic therapies targeting the VEGF pathway.

Author	Journal	Year	Phase	Patients	Outcome	Treatment	Target
Lee EQ et al. [27]	*Cancer*	2020	II	57	OS, PFS	Bevacizumab	VEGF
Mark R Gilbert et al. [36]	*J Neurooncol*	2017	II	60	OS, PFS	Bevacizumab	VEGF
Jeon JY et al. [42]	*Am J Neuroradiol*	2012	II	18	PFS	Bevacizumab	VEGF
Brown N et al. [38]	*PLoS One*	2016	II	38	OS, PFS	Cediranib	VEGFR
Hottinger AF et al. [28]	*Br J Cancer*	2014	I	17	OS, PFS	Sorafenib	VEGFR
Wetmore C et al. [29]	*Cancer Med*	2016	II	30	OS, PFS	Sunitinib	VEGFR
Kalpathy-Cramer J et al. [31]	*J Neurooncol*	2017	II	10	OS, PFS	Tivozanib	VEGFR
Broniscer A et al. [32]	*Clin Cancer Res*	2013	I	25	OS	Dasatinib and Vandetanib	VEGFR
Reardon DA et al. [40]	*Cancer*	2009	I	37	OS	Vatalanib	VEGFR
David A. Reardon [44]	*Cancer Chemother Pharmacol*	2012	I	64	PFS	AEE788	VEGFR

**Table 3:** Summary of the studies on HGG metabolic therapies targeting the HER pathway.

Author	Journal	Year	Phase	Patients	Outcome	Treatment	Target
Michael W Ronellenfitsch et al. [30]	*Acta Neuropathol Commun*	2018	III	149	OS, PFS	Nimotuzumab	EGFR
Lv S et al. [39]	*Int J Oncol*	2012	II	35	OS, PFS	Cetuximab	EGFR
Ian F. Pollack et al. [45]	*Neuro Oncol*	2011	II	43	OS, PFS	Gefitinib	EGFR
Broniscer A et al. [32]	*Clin Cancer Res*	2013	I	25	OS	Dasatinib and Vandetanib	EGFR
David A. Reardon [44]	*Cancer Chemother Pharmacol*	2012	I	64	PFS	AEE788	ERBB2

**Table 4:** Summary of the studies on HGG metabolic therapies targeting the PDGF pathway.

Author	Journal	Year	Phase	Patients	Outcome	Treatment	Target
Hottinger AF et al. [28]	*Br J Cancer*	2014	I	17	OS, PFS	Sorafenib	PDGFR
Wetmore C et al. [29]	*Cancer Med*	2016	II	30	OS, PFS	Sunitinib	PDGFR
Broniscer A et al. [32]	*Clin Cancer Res*	2013	I	25	OS	Dasatinib and Vandetanib	PDGFR
Reardon DA et al. [40]	*Cancer*	2009	I	37	OS	Vatalanib	PDGFR

**Table 5:** Summary of the studies on HGG metabolic therapies targeting the mTOR pathway.

Author	Journal	Year	Phase	Patients	Outcome	Treatment	Target
Reardon DA et al. [33]	*J Neurooncol*	2010	II	32	OS, PFS	Erlotinib	mTOR
Ma DJ et al. [37]	*Neuro Oncol*	2015	II	100	OS, PFS	Everolimus	mTOR
Galanis E et al. [41]	*J Clin Oncol*	2005	II	65	OS	Temsirolimus	mTOR
Babak et al. [43]	*Neuro-Oncol*	2018	II	171	PFS	Everolimus	mTOR

**Table 6:** The modified NOS.

No.	Criterion	Decision Rule	Score (* = 1; no * = 0)
SELECTION			
1	Representativeness of the exposed cohort	(a) Consecutive eligible participants were selected, participants were randomly selected, or all participants were invited to participate from the source population; * (b) Not satisfying requirements in part (a), or not stated.	
2	Selection of the non-exposed cohort	(a) Selected from the same source population; * (b) Selected from a different source population; (c) No description.	
3	Ascertainment of exposure	(a) Medical record; * (b) Structured interview; * (c) No description.	
4	Demonstration that outcome of interest was not present at the start of the study	(a) Yes; * (b) No or not explicitly stated.	
COMPARABILITY			
1	Were there clearly defined inclusion and exclusion criteria?	(a) Yes; * (b) No or not explicitly stated.	
OUTCOME			
1	Assessment of outcome	(a) Independent or blind assessment stated, or confirmation of the outcome by reference to secure records; * (b) Record linkage (e.g., identified through ICD codes on database records); * (c) Self-report with no reference to original structured injury data or imaging; (d) No description.	
2	Was follow-up long enough for outcomes to occur?	(a) Yes (≥12 months); * (b) No (<3 months).	
3	Adequacy of follow up of cohorts	(a) Complete follow up—all participants accounted for; * (b) Subjects lost to follow up unlikely to introduce bias (<20% lost to follow up or description provided of those lost); * (c) Follow up rate <85% and no description of those lost provided; (d) No statement.	
SCORE			

## Data Availability

Data are available in a repository with public access.
